# Quantifying the Effects of Ethanol and Temperature on the Fitness Advantage of Predominant *Saccharomyces cerevisiae* Strains Occurring in Spontaneous Wine Fermentations

**DOI:** 10.3389/fmicb.2018.01563

**Published:** 2018-07-13

**Authors:** Donatella Ganucci, Simona Guerrini, Silvia Mangani, Massimo Vincenzini, Lisa Granchi

**Affiliations:** ^1^FoodMicroTeam, Academic Spin-Off of the University of Florence, Florence, Italy; ^2^Department of Management of Agricultural, Food and Forestry Systems (GESAAF), University of Florence, Florence, Italy

**Keywords:** *Saccharomyces cerevisiae* strains, spontaneous wine fermentation, fitness advantage, temperature, ethanol

## Abstract

Different *Saccharomyces cerevisiae* strains are simultaneously or in succession involved in spontaneous wine fermentations. In general, few strains occur at percentages higher than 50% of the total yeast isolates (predominant strains), while a variable number of other strains are present at percentages much lower (secondary strains). Since *S. cerevisiae* strains participating in alcoholic fermentations may differently affect the chemical and sensory qualities of resulting wines, it is of great importance to assess whether the predominant strains possess a “dominant character.” Therefore, the aim of this study was to investigate whether the predominance of some *S. cerevisiae* strains results from a better adaptation capability (fitness advantage) to the main stress factors of oenological interest: ethanol and temperature. Predominant and secondary *S. cerevisiae* strains from different wineries were used to evaluate the individual effect of increasing ethanol concentrations (0-3-5 and 7% v/v) as well as the combined effects of different ethanol concentrations (0-3-5 and 7% v/v) at different temperature (25–30 and 35°C) on yeast growth. For all the assays, the lag phase period, the maximum specific growth rate (μ_max_) and the maximum cell densities were estimated. In addition, the fitness advantage between the predominant and secondary strains was calculated. The findings pointed out that all the predominant strains showed significantly higher μ_max_ and/or lower lag phase values at all tested conditions. Hence, *S. cerevisiae* strains that occur at higher percentages in spontaneous alcoholic fermentations are more competitive, possibly because of their higher capability to fit the progressively changing environmental conditions in terms of ethanol concentrations and temperature.

## Introduction

Spontaneous grape juice fermentation into wine is carried out by the yeast populations naturally occurring on the grape surface and in the winery environment (Sabate et al., 2002; Bisson, 2012). In this process, in the vats filled at the beginning of the vintage, non-*Saccharomyces* yeast species usually predominate in the early stages and later, with ethanol increasing, they are replaced by *Saccharomyces cerevisiae* because of higher resistance of this yeast species to alcohol (Pretorius, 2000; Bisson, 2005; Querol and Fleet, 2006; Albergaria and Arneborg, 2016). This substitution may be explained by the competitive exclusion of the less efficient yeasts species (Arroyo-López et al., 2011). Although ethanol production has been the cause traditionally accepted for explaining the imposition of *S. cerevisiae* on non-*Saccharomyces* yeast species, other death-inducing mechanisms have been proposed as responsible for its competitive advantage, including the production of antimicrobial compounds, such as SO_2_ and peptides, the cell-to-cell contact, and the temperature increase during alcoholic fermentation (Goddard, 2008; Salvadó et al., 2011; Perrone et al., 2013; Branco et al., 2015; Williams et al., 2015; Albergaria and Arneborg, 2016; Pérez-Torrado et al., 2017). Therefore, as the fermentation progresses, the grape must becomes a more selective environment representing a highly specialized ecological niche (Salvadó et al., 2011). Nevertheless, *S. cerevisiae* populations generally display a high polymorphism in spontaneous wine fermentations. Indeed, numerous studies, carried out by molecular techniques on the population dynamics of *S. cerevisiae* during spontaneous wine fermentations in several regions all over the world, have established that different strains are simultaneously or in succession involved during the whole fermentation process (Querol et al., 1994; Pramateftaki et al., 2000; Augruso et al., 2005; Schuller et al., 2005; Agnolucci et al., 2007; Csoma et al., 2010; Orlić et al., 2010; Capece et al., 2011, 2012; Mercado et al., 2011; Bisson, 2012). In some cases *S. cerevisiae* strains were able to dominate the alcoholic fermentation in all vats of the same winery, independently of the grapevine cultivar (Frezier and Dubourdieu, 1992; Guillamón et al., 1996), whereas other times the yeast strains were found to be specific for each grape variety (Blanco et al., 2006). In general, few *S. cerevisiae* strains occur at higher percentages (more than 30–50% of the total yeast isolates) while a variable number of strains are present at lower percentages. Therefore, these strains can be differentiated in “predominant” and “secondary” strains, respectively (Versavaud et al., 1995). In addition, the predominant strains can sometimes persist in alcoholic fermentations carried out in the same winery in consecutive years and can be described as “recurring” strains (Gutièrrez et al., 1999; Bisson, 2012). Since *S. cerevisiae* strains, participating in alcoholic fermentations, may differently affect the chemical and sensory qualities of resulting wines (Fleet, 2003; Romano et al., 2003; Villanova and Sieiro, 2006; Lopandic et al., 2007; Barrajón et al., 2011; Knight et al., 2015; Bokulich et al., 2016; Callejon et al., 2016), it is of great importance to assess whether the predominant strains retain the dominant behavior after their isolation from grape must fermentations. Furthermore, it could be noteworthy to investigate whether the predominance of these *S. cerevisiae* strains on others results from a different adaptation capability (fitness advantage) to some stress factors of oenological interest. Recently, two studies concerning competition between strains of *S. cerevisiae* species suggest that the dominance of one strain over another is dependent on the different SO_2_ production and resistance and on the cell-to-cell contact in mixed cultures, i.e., in the same environment (Perrone et al., 2013; Pérez-Torrado et al., 2017). With the exception of studies on killer factors (Jacobs and van Vuuren, 1991; Pérez et al., 2001), to our knowledge, other surveys on the competition among strains of *S. cerevisiae* species are lacking. Considering that temperature and ethanol during alcoholic fermentation are held responsible for the ability of *S. cerevisiae* to dominate on non-*Saccharomyces* yeasts or on other *Saccharomyces* spp. (Goddard, 2008; Williams et al., 2015; Alonso del-Real et al., 2017; Henriques et al., 2018), the adaptability to these two factors could be also involved in determining the dominance of predominant on secondary *S. cerevisiae* strains. Moreover, the predominance of *S. cerevisiae* strains with particular resistance capability to these two stress factors could contribute to the construction of an ecological niche typical of each fermentation tank and possibly winery.

Therefore, the aim of this study was to investigate whether the predominance of some *S. cerevisiae* strains during spontaneous alcoholic fermentation results from a better adaptation capability of these strains to ethanol and temperature as stress factors. At first, dynamics of *S. cerevisiae* strains during spontaneous wine fermentations carried out in six Tuscan wineries were monitored to identify one predominant and one secondary *S. cerevisiae* strains from each winery. After that, the predominant and secondary strains of each winery were tested in synthetic media to compare their growth capability when subjected to stress of ethanol and temperature. Finally, the fitness advantage (as defined by Salvadó et al., 2011) was calculated to verify if the predominant strains owned a better adaptation capability than the secondary strains to the two main stress factors of oenological interest.

## Methods

### Isolation of *Saccharomyces cerevisiae* from spontaneous wine fermentations

Spontaneous wine fermentations were carried out under industrial conditions during the same vintage in six wineries (A, B, C, D, E, and F) producing DOC and DOCG red wines in Tuscany region (Central Italy). In all the winery except the E, commercial starter yeasts were never used. In each winery, various fermentation tanks (6 in winery A; 2 in B; 8 in C; 6 in D; 4 in E; 6 in F) were filled with musts from different grape varieties (S: *Sangiovese*; CA: *Cabernet*; N: *Pinot Nero*; M: *Merlot*; V: *Vermentino*). Yeasts were isolated by plating the must/wine samples on WL Nutrient Agar medium (Oxoid Ltd, Basingstoke, Hampshire, UK) containing sodium propionate (2 g/L) and streptomycin (30 mg/L) to inhibit mold and bacterial growth, respectively. Plates were incubated for 48 h at 30°C, under aerobic conditions. *S. cerevisiae* isolates were identified by PCR-RFLP analysis of the rDNA Internal Transcribed Spacer (ITS) according to Esteve-Zarzoso et al. (1999).

About 25 isolates from each fermentation tank belonging to *S. cerevisiae* species were stored in liquid cultures containing 50% (v/v) glycerol at −80°C until use.

### Genotypic characterization of *S. cerevisiae* isolates

Genotypic differentiation of *S. cerevisiae* isolates was performed by mitochondrial DNA restriction analysis (mtDNA-RFLP) and the restriction endonucleases *Rsa*I and *Hinf* I (Granchi et al., 2003). The restriction DNA fragments were separated on 0.8%(w/v) agarose gels containing ethidium bromide (1 mg mL^−1^) by electrophoresis in 1X-TBE buffer (90 mMTris-borate, 2 mM, EDTA pH 8.0) at 4 Vcm^−1^ for 6 h. The RFLP patterns were submitted to pairwise comparison using the Dice coefficient (SD) (Sneath and Sokal, 1973) and cluster analysis with unweighted pair group method (UPGMA) by Gel Compar 4.0 software (Applied Math, Kortrijk, Belgium). *S. cerevisiae* diversity in each winery was quantified by using the two indices “H” and “e” as proposed by Shannon–Weaver (Shannon and Weaver, 1963).

### Laboratory-scale fermentations to verify the predominance behavior of *S. cerevisiae* strains

The medium used for laboratory scale fermentation was the chemically defined grape juice medium reported in the Table 1 of the RESOLUTION OIV-OENO 370 (2012). The synthetic medium was buffered to pH 3.3 using HCl 1N and sterilized by filtration. Fermentation experiments were carried out in triplicate in 250-mL Erlenmeyer flasks containing 160 mL of the medium. Each flask was inoculated with two *S. cerevisiae* strains at the same concentration (10^4^ cells mL^−1^) from pre-cultures grown for 24 h in the same medium. After inoculation, the flasks were sealed with a Müller trap previously filled with sulphuric acid to allow only CO_2_ to outflow and they were incubated at 28°C. The fermentation progress was followed by determining the weight loss due to CO_2_ release until the weight remained constant. At the end of fermentation, chemical analysis were performed by HPLC (Schneider et al., 1987; Granchi et al., 1998). Viable counts of the yeasts were performed, after 24 h and 10 days from inoculation, on WL Nutrient Agar medium (Oxoid Ltd, Basingstoke, Hampshire, UK) incubated 48 h at 30°C in aerobic conditions. To calculate isolation frequencies of the two *S. cerevisiae* strains inoculated together in the each fermentation flask, a significant number of colonies from WL Nutrient Agar medium were assayed using mtDNA-RFLP as reported above.

**Table 1:** Isolation frequencies, expressed as percentages, of mt-DNA profiles of *S. cerevisiae* from 32 spontaneous wine fermentations carried out in different tanks during the same vintage in six wineries in Tuscany (Italy).

**Sample code**	**mt-DNA profiles**	**Isolation frequency (%)**
AS1	**AIV**- AV - AVI - AVII - AXII - AXIII	**46** - 17 - 17 - 4 - 8 - 8
AS2	**AIV** - AV - AVI - AVII - AXII - AXIII	**29** - 8 - 21 - 17 - 12.5 - 12.5
AM1	AI - AIV - AV - AVII - **AX** - **AXII** - AXIV	4 - 4 - 13 - 13 - **36** - **26** - 4
AM2	AVI - AVII - **AX** - AXIV	4 - 12.5 - **79.5** - 4
AP1	**AX** - AXI	**90** - 10
AP2	AIV - AX - AXI - **AXII** - AXIII	16.5 - 16.5 - 8.5 - **42** - 16.5
BS1	BI - **BII** - BIII - **BIV** - BVII - BVIII - BIX	10 - **35** - 5 - **35** - 5 - 5 - 5
BS2	BII - BIV - BV - BVI - BVII - BVIII - BX BXI - BXII - BXIII - BXIV - BXV - BXVI	20.2−3.8 - 3.8−15.2 - 11.4−7.6−3.8 3.8 - 15.2 - 3.8 - 3.8 - 3.8 - 3.8
CS1	CI - CII - CIII - CIV - CVI - **CVII**	10 - 5 - 10 - 25 - 5 - **45**
CS2	CI - CII - **CIII** -CIV - CV - CVII	10 - 5 - **60** - 5 - 5 - 15
CS3	CI - **CIII** - CIV - CV	10.6 - **73.5** - 10.6 - 5.3
CS4	CI - **CIII**	10 - **90**
CM1	CI - **CIII**	10 - **90**
CM2	CI - **CIII**	5 - **95**
CM3	CI - **CIII**	16 - **84**
CM4	CI - **CIII**	5 - **95**
DS1	DI - DIV - DVI - DX	4.2 - 83.2 - 4.2 - 8.4
DS2	**DIV** - DVI - DVII	**69.6** - 17.4 - 13
DS3	DI - DII - **DIV** - DVI - DVII - DXI	12.5 - 4.2 - **41.6** - 12.5 - 25 - 4.2
DS4	DI - DIII - **DIV**- DVI -DVII - DVIII	22.5 - 9 - **32.5** - 13.5−18 - 4.5
DM1	**DI** - DII - DIII - DIV - DV	**50** - 5 - 5 - 35 - 5
DM2	DI - DIII - **DIV** - DVI - **DVII** - DIX	4.25 - 4.25 - **46** - 4.25 - **37** - 4.25
ES1	**EI** - **EVII**	**40** - **60**
EC2	**EI** - EIII - EVII	**71.5** - 9.5 - 19
EM1	**EI** - EII - EIII - EIV - EV - EVI	**68.5** - 14 - 7 - 3.5 - 3.5 - 3.5
EM2	**EI** - EIII	**96** - 4
FVN1	FI - **FI****I** - FIII - FIV - FV	**42** - 8 - 8 - **34** - 8
FVN2	FI - **FII** - FIV	**60** - 10 - **30**
FVN3	FI - **FIII** - FIV - FVII - FVIII	**44** - 25 - 19 - 6 - 6
FVB1	FI - **FIII** - FIV - FVII	13.5 - **40** - **40** - 6.5
FVB2	FI - **FIII** - FIV	6.5 - **53.5** - **40**
FVB3	FI - FII - FIII - **FIV** - FV	13.5 - 6.5 - 26.5 - **47** - 6.5

*The sample codes indicate the winery (A, B, C, D, E and F), the grape variety (Sangiovese: S, Cabernet: C, Pinot Nero: P, Merlot: M, Vermentino nero: VN and Vermentino bianco: VB), and the number of the fermentation tank. The predominant S. cerevisiae strains are in bold and underlined*.

### Effect of ethanol on the *S. cerevisiae* growth

The medium used to assay the effect of ethanol on the growth of the different *S. cerevisiae* strains was Yeast Nitrogen Base (Difco) integrated with glucose (20 g L^−1^) and increasing concentrations of ethanol (0-3-5 and 7% v/v). Fermentation trials were carried out at 28°C in triplicate in 100-mL Erlenmeyer flasks containing synthetic medium (50 mL each flask) inoculated with 2 × 10^6^ cells mL^−1^ (axenic cultures) from pre-cultures of the various *S. cerevisiae* strains grown for 24 h in the same medium without ethanol. Fermentation progress was monitored every 2 h quantifying by HPLC sugar degradation (Lefebvre et al., 2002). On the same samples, viable cells were determined by Thoma counting chamber and fluorescence microscopy to monitor the yeast growth as reported by Granchi et al. (2006). The decimal logarithms of viable counts detected during the time course of each fermentation after 8 and 24 h were fitted both to Baranyi and Roberts (1994) function and to reparametrized Gompertz equation proposed by Zwietering et al. (1990) by using Combase-DMfit software and GraphPadPrism 5, respectively. Finally, the area under the growth curve of each strain was calculated as reported by Arroyo-López et al. (2009, 2010) and Castilleja et al. (2017), using GraphPadPrism 5.

### Combined effect of ethanol and temperature on *S. cerevisiae* growth

To evaluate the combined effect of temperature and ethanol on the *S. cerevisiae* growth, a Box-Wilson Central Composite Design with two variables and three levels was used. The medium used was Yeast Nitrogen Base (Difco) integrated with 20 g mL^−1^. The range of temperatures was 25–35°C, while the range of ethanol was 0–7% (v/v). As reported in the previous experiment, fermentation progress was monitored every 2 h (for 24 h) quantifying by HPLC the sugar degradation, while the viable yeast cells were determined by Thoma counting chamber and fluorescence microscopy. The decimal logarithms of viable yeast cells detected during the time course of each fermentation were fitted to Gompertz function using GraphPadPrism 5. The area under the growth curve of each strain was calculated using GraphPadPrism 5.

## Results

### Predominant *S. cerevisiae* strains in spontaneous wine fermentations

**Figure 1:**
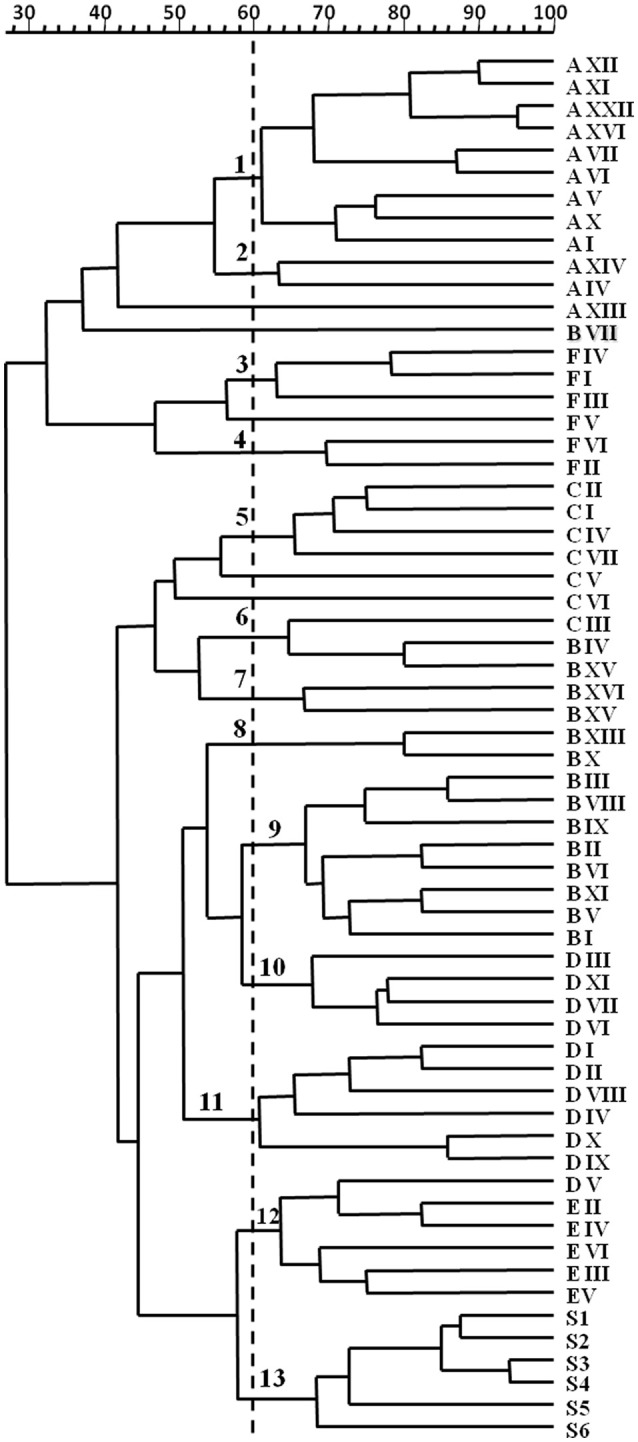
Dendrogram from UPGMA clustering analysis, based on Dice coefficient of mt-DNA *Rsa*I restriction patterns of the *S. cerevisiae* isolates from 32 spontaneous wine fermentations carried out in six different wineries (A, B, C, D, E, and F) in Tuscany (Italy). S1-S6 indicate commercial starter cultures. Arabic numerals at 60% similarity indicate the different clusters.

Dynamics of yeasts in spontaneous wine fermentations carried out during the same vintage in six wineries producing DOC and DOCG red wines in Tuscany region (Italy) were monitored. For each winery, from two to eight fermentation tanks were filled with musts obtained from different grape variety (*Sangiovese, Cabernet, Pinot Nero, Merlot, Vermentino*), and allowed to ferment naturally. When the yeast population reached the maximum growth yield, 637 isolates belonging to *S. cerevisiae* species (about 25 isolates from each fermentation tank) were analyzed by mitochondrial DNA (mt-DNA) restriction analysis. The mt-DNA profiles obtained for each tank in the different wineries and the relative frequencies of isolation expressed as percentages are reported in table 1. Results revealed that, independently of the winery and the grape variety, each spontaneous wine fermentations was carried out by one or two predominant *S. cerevisiae* strains at high frequency, ranging from about 30–90%, in association with a variable number of secondary strains at low frequency. Moreover, some predominant strains were shared by different grape varieties fermented in various tanks (Table 1). By calculating the isolation frequency of each different mt-DNA profile occurring in each winery, a total of 58 *S. cerevisiae* strains out 637 isolates from the six wineries were obtained (Table 2). Then, they were distributed in three frequency classes: strains at low frequency (<10%), strains at frequency ranging from 10 to 30% and predominant strains at frequency>30% (Table 3). Although according to Shannon's index “*H*,” estimating the richness of *S. cerevisiae* strains found in the six wineries, different diversity level was observed, only one *S. cerevisiae* strain emerged as clearly predominant in each winery except for the cellar B. Indeed, the evenness index “*e*,” ranging between 0 and 1 and that increases with the decreasing of the number of isolates showing the same mt-DNA, assumed the highest value in the winery B in which the predominant strain occurred at the lowest frequency value found. All the mt-DNA profiles corresponding to the different *S. cerevisiae* strains were also analyzed using UPGMA clustering analysis and the resulting dendrogram is reported in Figure 1. In this elaboration were also included the mt-DNA profiles of six commercial starter strains most commonly used in Tuscany. The *S. cerevisiae* strains, at 60% of similarity, grouped into 13 clusters mainly based on the winery where they come from, independently on the grape variety used. In particular, the *S. cerevisiae* strains isolated from the winery B were included in the clusters 6-7-8 and 9, while the commercial starter strains grouped in the same cluster.

**Table 2:** Distribution of different mt-DNA profiles of *S. cerevisiae* in the six wineries (A, B, C, D, E, and F) in the same vintage.

**Winery code**											
**A**		**B**		**C**		**D**		**E**		**F**	
**mt-DNA profiles**	**Isolation frequency (%)**	**mt-DNA profiles**	**Isolation frequency (%)**	**mt-DNA profiles**	**Isolation frequency (%)**	**mt-DNA profiles**	**Isolation frequency (%)**	**mt-DNA profiles**	**Isolation frequency (%)**	**mt-DNA profiles**	**Isolation frequency (%)**
AI	1	BI	4.3	CI	9.5	DI	14.5	EIV	1.0	FI	25
AIV	18	BII	**26**	CII	2.0	DII	1.5	EII	4.0	FII	3
AV	7.5	BIII	2.2	CIV	5.0	DIII	3.0	EIII	5.0	FIII	29
AVI	8.5	BIV	17.4	CIII	**74**	DIV	**52**	EI	**71**	FIV	**36**
AVII	9.5	BV	2.2	CV	1.3	DV	0.7	EV	1.0	FV	2
AX	**32**	BVI	8.7	CVI	0.7	DVI	8.7	EVI	1.0	FVII	3
AXI	2	BVII	8.7	CVII	7.5	DVII	16	EVII	17	FVIII	2
AXII	14	BVIII	6.5			DVIII	0.7				
AXIII	6	BIX	2.2			DIX	0.7				
AXIV	1.5	BX	2.2			DX	1.5				
		BXI	2.2			DXI	0.7				
		BXII	8.7								
		BXIII	2.2								
		BIV	2.2								
		BXV	2.2								
		BXVI	2.2								

*Predominant strains are in bold and underlined*.

**Table 3:** Number of mt-DNA profiles of *S. cerevisiae* at different isolation frequency in the six wineries and related indices Shannon and Weaver (1963).

**Winery**	**Frequency (%)**			***H***	***e***
	**<10**	**10–30**	**>30**		
A	7	2	1	1.86	0.81
B	14	2	-	2.35	0.85
C	6	-	1	1.02	0.49
D	8	2	1	1.73	0.61
E	5	1	1	1.01	0.46
F	4	2	1	1.44	0.74

*H, biodiversity index; e, evenness*.

In conclusion, according to these results, for each winery one predominant strain, indicated by the code HF (High Frequency), and one secondary strain, indicated by the code LF (Low Frequency), were chosen with the aim to compare their behavior in subsequent trials. The HF-*S. cerevisiae* strains displayed the following mt-DNA profiles: AX – BII – CIII – DIV – EI and FIV, while the LF-*S. cerevisiae* strains corresponded to the mt-DNA profiles AI – BI – CVI – DXI – EVI and FV.

### Laboratory scale fermentation to verify the predominance behavior of HF-*S. cerevisiae* strains on LF-*S. cerevisiae* strains

**Figure 2:**
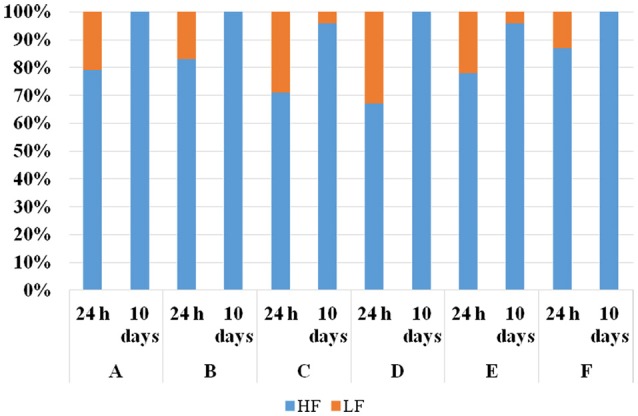
Isolation frequencies of one “high frequency”(HF)-*S. cerevisiae* strain and one “low frequency ” (LF) *S. cerevisiae* strain, representative of each winery (A, B, C, D, E and F) after 24 h and 10 days in co-fermentations in synthetic must at 28°C. The “HF” and “LF” strains were inoculated at the same cell concentration (10^4^ cell/mL).

To verify whether the *S. cerevisiae* strains identified as HF were actually able to dominate on the strains identified as LF, laboratory-scale co-fermentations were performed. One HF and one LF strain isolated from each winery were co-inoculated in synthetic must at the same cell concentration (10^4^ CFU/mL). This value was chosen in order to simulate the low *S. cerevisiae* cell densities usually found in spontaneous alcoholic fermentation. Co-fermentations carried out at 28°C by the strains from the wineries A, C, D, and F were completed in about 10 days, even if the strains from the wineries D and F showed lower fermentation rates than those from the wineries A and C (data not shown). On the contrary, the strains from winery B were unable to complete alcoholic fermentation (20% w/v of reducing sugars). During the fermentations, samples were taken at two different times (after 24 h and 10 days from the inoculation) in order to assess mt-DNA patterns of the *S. cerevisiae* isolates as well as their isolation frequencies. Figure 2 shows the isolation frequencies of HF and LF strains assayed for each fermentation after 24 h and 10 days from the inoculation. Although the starting inoculum of HF and LF strains was at the same cell concentration, after 24 h the isolation frequencies of the LF strains were lower than 35% in all the fermentations. After 10 days the HF strains isolated from A, B, D, and F winery showed isolation percentages of 100%, while HF strains from C and E of 96%. Therefore, the results demonstrated that during the fermentative process all the HF-*S. cerevisiae* strains occurred progressively at higher percentages demonstrating to retain in laboratory the “predominance behavior” displayed in industrial fermentations.

### Effect of ethanol on the growth performance and the fitness advantage of HF and LF-*S. cerevisiae* strains

The HF and LF-*S. cerevisiae* strains of the experiment previously described were also used to perform axenic fermentations in synthetic media containing various concentrations of ethanol (0-3-5 and 7% v/v). The aim of these trials was to investigate on the growth performance and on the fitness of HF and LF-strains, in order to detect any behavior justifying the different isolation frequencies observed during the spontaneous fermentations. Baranyi and Roberts-model was used to estimate the fermentative performance of the strains in terms of lag phase (λ), maximum specific growth rate (μmax) and maximum cell densities at the end of fermentations (Table 4). The goodness of fit of this model was appropriate for all the strains assayed, R^2^ values being, higher than 0.90 (data not shown). The findings pointed out that the μ_max_ values of HF- strains from each winery were significantly higher than the μ_max_ values of the LF-strains, at least in the presence of one of the ethanol concentrations considered. In particular, the HF-strains coming from A, B, E, and F wineries showed higher values in the presence of 5% ethanol, while HF-strains from wineries C and D, in the presence of 7 and 3% ethanol, respectively. Moreover, HF-strains from five wineries (A, C, D, E, and F) showed a higher growth yield than the respective LF-strains in synthetic medium containing 3 or 5% ethanol, indicating a higher alcohol tolerance of the HF-strains than LF-*S. cerevisiae* strains. On the contrary, only the HF-strains from the winery C and F showed a lag phase shorter than that of the respective LF-strains, when ethanol concentration was 3 or 5% (Table 4). To assess the overall effect of ethanol on the HF and LF-strains from each winery, the inhibition percentages of the growth due to ethanol was estimated comparing the area under the growth curve of the positive control (absence of ethanol) with the areas of the other conditions (presence of ethanol at different concentrations: 3, 5, and 7%). Therefore, for each strain, the percentage of inhibition determined by the different ethanol concentrations was calculated using the following formula: = [1 – (Area under the growth curve in presence of ethanol/Area under the curve without ethanol)]^*^100 (Table 4). This parameter is shown to be inversely related to the lag phase and linearly related to both the maximum exponential growth rate and maximum cell densities reached and thus is appropriate to assess the overall yeast growth (Arroyo-López et al., 2010). HF-*S. cerevisiae* strains isolated from four winery (A, C, E, and F) showed an inhibition percentages significantly lower than the LF-strains coming from the same wineries at all the concentrations of ethanol tested, while in the case of remaining two wineries, B and D, this difference was observed only at 5 and 3% of ethanol, respectively.

**Table 4:** Growth parameters and inhibition percentages to ethanol of the HF and LF-*S. cerevisiae* strains in synthetic media at different ethanol concentrations.

**Ethanol (% v/v)**	**Lag phase (h)**		μ **(h**^**−1**^**)**		(cell/mL)^*^10^**6**^		**Inhibition percentages to ethanol**	
	**HF**	**LF**	**HF**	**LF**	**HF**	**LF**	**HF**	**LF**
**Winery A**								
0	2.858 ± 0.442	2.705 ± 0.389	0.179 ± 0.003	0.164 ± 0.005	12.00 ± 0.25	11.75 ± 0.15	–	–
3	3.288 ± 0.237	4.349 ± 0.593	0.162 ± 0.011	0.149 ± 0.002	10.19 ± 0.06^S^	8.69 ± 0.06	16.99 ± 1.72^S^	36.00 ± 0.63
5	4.796 ± 0.274	4.187 ± 0.555	0.158 ± 0.008^S^	0.058 ± 0.013	7.93 ± 0.18^S^	3.37 ± 0.12	47.66 ± 1.68^S^	76.93 ± 2.13
7	4.989 ± 0.076	n.f.^*^	0.044 ± 0.035	n.f.^*^	2.68 ± 0.06	2.12 ± 0.12	66.23 ± 4.56^S^	96.42 ± 2.07
**Winery B**								
0	2.763 ± 0.309	2.523 ± 0.523	0.173 ± 0.007	0.176 ± 0.001	15.70 ± 0.50	15.10 ± 0.10	–	–
3	3.763 ± 0.498	3.782 ± 0.727	0.179 ± 0.020	0.155 ± 0.029	11.70 ± 0.50	9.10 ± 0.50	32.69 ± 9.61	32.24 ± 5.70
5	4.086 ± 0.287	3.765 ± 0.230	0.114 ± 0.002^S^	0.097 ± 0.003	5.60 ± 0.01^S^	4.80 ± 0.01	54.12 ± 0.48^S^	58.04 ± 0.42
7	4.665 ± 2.423	5.451 ± 0.390	0.073 ± 0.049	0.045 ± 0.019	3.00 ± 0.10	2.60 ± 0.10	83.54 ± 3.50	89.63 ± 0.22
**Winery C**								
0	2.317 ± 0.185	1.751 ± 0.071	0.181 ± 0.010	0.159 ± 0.005	19.90 ± 0.70	17.25 ± 0.75	–	–
3	2.142 ± 0.059^S^	3.249 ± 0.063	0.137 ± 0.001	0.135 ± 0.005	11.35 ± 0.15^S^	9.32 ± 0.42	23.43 ± 1.02^S^	46.83 ± 3.85
5	3.243 ± 0.254	3.130 ± 0.202	0.122 ± 0.011	0.090 ± 0.012	7.45 ± 0.05^S^	5.30 ± 0.20	47.98 ± 4.79^S^	61.13 ± 2.10
7	4.176 ± 0.774	4.236 ± 0.822	0.044 ± 0.006^S^	0.025 ± 0.001	3.50 ± 0.25	2.50 ± 0.10	66.64 ± 0.96^S^	88.61 ± 4.52
**Winery D**								
0	1.659 ± 0.036	1.501 ± 0.189	0.156 ± 0.005	0.143 ± 0.001	17.75 ± 0.35^S^	15.63 ± 0.12	–	–
3	1.920 ± 0.211	1.479 ± 0.074	0.142 ± 0.001^S^	0.112 ± 0.003	14.55 ± 0.45^S^	11.50 ± 0.10	14.80 ± 0.46^S^	19.00 ± 0.78
5	1.936 ± 0.001	1.921 ± 0.352	0.077 ± 0.004	0.068 ± 0.005	6.93 ± 0.31	6.15 ± 0.25	40.47 ± 0.42	43.39 ± 2.08
7	1.982 ± 0.031	1.995 ± 0.144	0.049 ± 0.006	0.045 ± 0.005	4.25 ± 0.25	3.75 ± 0.15	63.89 ± 3.46	73.62 ± 1.26
**Winery E**								
0	2.568 ± 0.008	2.341 ± 0.201	0.169 ± 0.001	0.169 ± 0.006	17.90 ± 0.30	18.55 ± 0.25	–	–
3	3.677 ± 0.005	3.706 ± 0.460	0.153 ± 0.001^S^	0.102 ± 0.002	9.44 ± 0.19^S^	5.57 ± 0.32	37.06 ± 1.17^S^	60.19 ± 2.06
5	3.346 ± 0.257	4.062 ± 0.717	0.156 ± 0.010^S^	0.077 ± 0.009	7.15 ± 0.20^S^	3.87 ± 0.12	59.05 ± 1.81^S^	78.74 ± 4.43
7	4.042 ± 0.077	5.124 ± 1.200	0.038 ± 0.001^S^	0.016 ± 0.001	2.87 ± 0.12^S^	2.19 ± 0.06	82.39 ± 1.17^S^	96.30 ± 2.34
**Winery F**								
0	0.340 ± 0.210^S^	1.436 ± 0.223	0.136 ± 0.005	0.136 ± 0.004	18.00 ± 0.10	17.40 ± 0.40	–	–
3	0.790 ± 0.236^S^	1.878 ± 0.023	0.123 ± 0.006	0.117 ± 0.001	13.00 ± 0.40^S^	9.37 ± 0.37	19.17 ± 3.22^S^	35.52 ± 3.20
5	1.342 ± 0.144^S^	2.464 ± 0.090	0.097 ± 0.004^S^	0.075 ± 0.007	7.81 ± 0.18^S^	5.14 ± 0.24	45.80 ± 1.50^S^	63.86 ± 0.10
7	4.175 ± 0.306	4.670 ± 0.744	0.105 ± 0.004^S^	0.055 ± 0.007	5.14 ± 0.24^S^	2.96 ± 0.16	77.30 ± 0.38^S^	88.77 ± 3.48

The growth parameters were calculated using Baranyi and Roberts-model (Combase DMfit software), the inhibition percentages to ethanol were calculated using the following formula: = [1- (Area under the growth curve in presence of ethanol/Area under the curve without ethanol)]^*^ 100. All the results are expressed as mean ± standard deviation; S, significant different (t-Test; p < 0.05); n.f., no significant fit.

**Figure 3:**
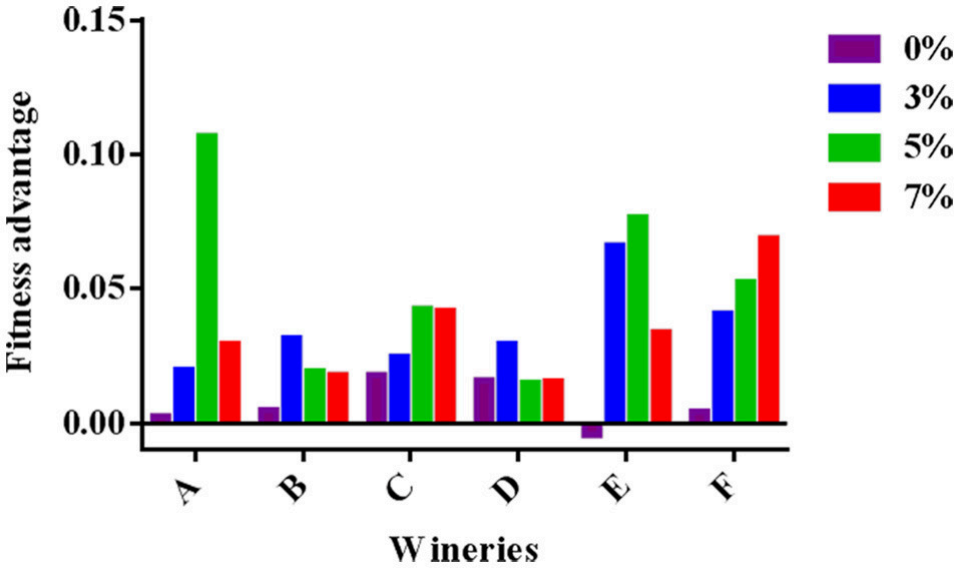
Fitness advantage at different ethanol concentrations calculated for each pair of HF/LF-*S. cerevisiae* strains from the six different wineries (A, B, C, D, E, and F) considering the average growth rate calculated between 0 and 8 h in synthetic medium at 28°C.

Finally, to quantify how increasing ethanol concentrations affects competition between HF- and LF-strains isolated from each winery, the concept of fitness advantage (Goddard, 2008; Salvadó et al., 2011) was used. Two main factors affect the yeast fitness: the maximum specific growth rate (μ_max_) and the duration of the lag phase (Buchanan and Solberg, 1972; Swinnen et al., 2004; Oxman et al., 2008). Therefore, in order to consider both factors, the fitness advantage was calculated taking into account the average growth rate (v) between 0 and 8 h using the following mathematical formula: fitness advantage (*m*) = v HF – v LF. In Figure 3, the data obtained at different ethanol concentrations, ranging from 0.01 to 0.11 (h^−1^), are shown. Independently on ethanol concentration, values resulted positive, demonstrating the fitness advantage of HF-*S. cerevisiae*—strains.

### Combined effect of temperature and ethanol on the growth performance and the fitness advantage of HF and LF-*S. cerevisiae* strains

Temperature and ethanol can considerably affect yeast growth and thus the wine fermentation kinetics. The contemporary presence of these two factors could play an important role in niche construction favoring some strains of *S. cerevisiae* compared to others in wine fermentation. To prove this selective effect, the combined effect of these two parameters on HF and LF-strains was studied in laboratory scale fermentation planning the experiment according to a central composite design with two variable (ethanol and temperature) and three-level. In particular, the three conditions of temperatures were 25, 30, and 35°C, while the three concentrations of ethanol were 0, 3.5, and 7%, obtaining nine combinations in total. Gompertz model was used to estimate the performances of the strains in terms of lag phase period, maximum specific growth rate (μ_max_) and maximum cell densities of the various fermentation kinetics (Table 5). The goodness of fit of this model was appropriate for all the strains assayed, R^2^ values being, higher than 0.95 (data not shown). The comparison between the growth performances of each pair of strains representative of the six wineries showed that, when significant differences occurred they were always in favor of HF instead of LF strains (shorter lag phase, higher maximum specific growth rate, higher maximum cell densities). Similarly, the inhibition percentages due to the combined effect of ethanol and temperature, when differences were statistically significant, were always higher for the LF strains compared to the HF-strains.

**Table 5:** Growth parameters and inhibition percentages to ethanol of the HF and LF-*S. cerevisiae* strains in synthetic media at different ethanol concentrations.

**Ethanol (% v/v)**	**Temperature (°C)**	**Lag phase (h)**		μ **(h**^**−1**^**)**		**C (Log cell/mL)**		**Inhibition percentages**	
		**HF**	**LF**	**HF**	**LF**	**HF**	**LF**	**HF**	**LF**
**Winery A**									
0	25	1.173 ± 0.004^S^	1.478 ± 0.058	0.1478 ± 0.0001	0.1493 ± 0.0027	1.429 ± 0.001	1.444 ± 0.002	–	–
3.5	25	1.094 ± 0.014^S^	2.060 ± 0.056	0.1028 ± 0.0001^S^	0.0810 ± 0.0001	1.240 ± 0.001	1.245 ± 0.014	26.14 ± 0.24^S^	49.12 ± 1.08
7	25	2.468 ± 0.044^S^	5.100 ± 0.356	0.0646 ± 0.0009	0.0643 ± 0.0015	1.183 ± 0.011	1.165 ± 0.0034	64.98 ± 0.14^S^	83.37 ± 1.82
0	30	1.004 ± 0.071^S^	1.841 ± 0.040	0.1861 ± 0.0027	0.2081 ± 0.0075	1.331 ± 0.006^S^	1.280 ± 0.002	–	–
3.5	30	1.040 ± 0.052^S^	1.886 ± 0.116	0.1028 ± 0.0007^S^	0.08164 ± 0.0019	1.236 ± 0.005^S^	1.132 ± 0.006	34.13 ± 0.54^S^	56.22 ± 0.05
7	30	1.705 ± 0.062^S^	3.576 ± 0.007	0.0656 ± 0.0002^S^	0.04307 ± 0.0007	0.677 ± 0.001^S^	0.498 ± 0.004	66.33 ± 1.04^S^	87.11 ± 2.57
0	35	0.248 ± 0.041^S^	1.732 ± 0.136	0.1245 ± 0.0015^S^	0.1650 ± 0.0075	1.295 ± 0.004^S^	1.137 ± 0.002	–	–
3.5	35	1.040 ± 0.053^S^	1.886 ± 0.016	0.06763 ± 0.0005^S^	0.0607 ± 0.0009	0.813 ± 0.012^S^	0.734 ± 0.002	48.38 ± 0.55	48.98 ± 0.07
7	35	1.463 ± 0.302	2.798 ± 0.456	0.03562 ± 0.0019	0.0300 ± 0.0026	0.321 ± 0.021	0.184 ± 0.006	79.96 ± 0.57^S^	87.55 ± 1.11
**Winery B**									
0	25	1.685 ± 0.010	1.643 ± 0.086	0.1862 ± 0.0045	0.2073 ± 0.0055	1.264 ± 0.002^S^	1.232 ± 0.004	–	–
3.5	25	1.642 ± 0.224	1.522 ± 0.319	0.0920 ± 0.0002^S^	0.0797 ± 0.0006	1.135 ± 0.012^S^	1.030 ± 0.0082	45.06 ± 0.20^S^	54.63 ± 0.88
7	25	2.698 ± 0.115^S^	4.654 ± 0.044	0.0515 ± 0.0002	0.0574 ± 0.0018	1.065 ± 0.058	1.077 ± 0.209	68.60 ± 0.76	78.37 ± 2.96
0	30	1.324 ± 0.090	1.775 ± 0.050	0.2394 ± 0.0022	0.2296 ± 0.0041	1.240 ± 0.006	1.246 ± 0.002	–	–
3.5	30	1.899 ± 0.068	2.172 ± 0.075	0.1552 ± 0.0030^S^	0.1419 ± 0.0023	1.116 ± 0.008^S^	0.982 ± 0.005	31.29 ± 0.76^S^	42.59 ± 0.46
7	30	1.960 ± 0.046^S^	2.760 ± 0.043	0.0459 ± 0.0050	0.0398 ± 0.0017	0.675 ± 0.012	0.609 ± 0.013	72.34 ± 1.73	72.45 ± 0.99
0	35	1.789 ± 0.066	1.635 ± 0.018	0.2360 ± 0.0145	0.2343 ± 0.0009	1.220 ± 0.018	1.238 ± 0.001	–	–
3.5	35	1.900 ± 0.378	3.065 ± 0.002	0.1336 ± 0.0087	0.1523 ± 0.0031	1.045 ± 0.020^S^	0.705 ± 0.011	39.00 ± 2.13^S^	58.31 ± 0.65
7	35	2.336 ± 0.075	3.140 ± 0.218	0.0223 ± 0.0010	0.0243 ± 0.0005	0.636 ± 0.119	0.451 ± 0.036	82.78 ± 0.66	84.66 ± 0.96
**Winery C**									
0	25	0.755 ± 0.039^S^	1.132 ± 0.072	0.1418 ± 0.0006	0.1464 ± 0.0032	1.453 ± 0.002^S^	1.321 ± 0.007	–	–
3.5	25	0.719 ± 0.067^S^	2.351 ± 0.214	0.1197 ± 0.0011	0.1306 ± 0.0066	1.345 ± 0.001^S^	1.147 ± 0.003	59.12 ± 1.09^S^	69.31 ± 0.52
7	25	1.129 ± 0.152^S^	15.140 ± 0.262	0.0880 ± 0.0030	0.0999 ± 0.0017	1.151 ± 0.001^S^	0.868 ± 0.067	84.79 ± 1.03^S^	92.52 ± 0.38
0	30	1.044 ± 0.143	1.023 ± 0.082	0.1571 ± 0.0042	0.1647 ± 0.0024	1.277 ± 0.001	1.267 ± 0.002	–	–
3.5	30	1.102 ± 0.027^S^	1.023 ± 0.002	0.1438 ± 0.0002^S^	0.1295 ± 0.0019	1.062 ± 0.009^S^	0.993 ± 0.004	47.88 ± 0.18^S^	55.32 ± 1.21
7	30	1.474 ± 0.090^S^	8.693 ± 1.092	0.0815 ± 0.0011^S^	0.0553 ± 0.0038	0.9602 ± 0.005	0.801 ± 0.269	82.25 ± 0.21^S^	93.73 ± 0.94
0	35	0.734 ± 0.101	0.830 ± 0.067	0.1616 ± 0.0042	0.1580 ± 0.0019	1.107 ± 0.009	1.128 ± 0.002	–	–
3.5	35	0.734 ± 0.174^S^	3.464 ± 0.237	0.1352 ± 0.0006	0.1382 ± 0.0083	1.041 ± 0.040	0.941 ± 0.001	85.97 ± 2.86	82.73 ± 2.01
7	35	0.818 ± 0.055^S^	7.352 ± 0.247	0.05821 ± 0.0003	0.0511 ± 0.0057	0.755 ± 0.001^S^	0.259 ± 0.015	94.72 ± 0.21	97.68 ± 0.02
**Winery D**									
0	25	2.983 ± 0.073^S^	2.436 ± 0.019	0.2043 ± 0.0027^S^	0.1592 ± 0.0020	1.254 ± 0.007	1.234 ± 0.016	–	–
3.5	25	4.631 ± 0.061	5.138 ± 0.207	0.1638 ± 0.0016	0.1349 ± 0.0111	0.992 ± 0.009	1.158 ± 0.045	33.92 ± 0.15^S^	51.12 ± 0.67
7	25	5.654 ± 0.129^S^	6.979 ± 0.029	0.0872 ± 0.0056	0.0867 ± 0.0078	0.990 ± 0.018	1.001 ± 0.030	74.40 ± 0.01	90.49 ± 4.57
0	30	2.108 ± 0.017^S^	2.225 ± 0.001	0.1905 ± 0.0020	0.1903 ± 0.0010	1.253 ± 0.001^S^	1.264 ± 0.002	–	–
3.5	30	3.156 ± 0.071	3.193 ± 0.027	0.1403 ± 0.0019^S^	0.1126 ± 0.0014	1.187 ± 0.011^S^	1.092 ± 0.011	43.33 ± 0.52^S^	55.47 ± 0.03
7	30	4.811 ± 0.049^S^	6.947 ± 0.193	0.0924 ± 0.0022	0.0801 ± 0.0073	0.927 ± 0.010	1.022 ± 0.034	84.52 ± 1.11	89.77 ± 1.63
0	35	2.692 ± 0.026^S^	2.467 ± 0.025	0.1849 ± 0.0011^S^	0.1639 ± 0.0015	1.229 ± 0.011^S^	1.090 ± 0.004	–	–
3.5	35	6.020 ± 0.036^S^	5.696 ± 0.016	0.0717 ± 0.0003^S^	0.0601 ± 0.0001	1.013 ± 0.046	1.449 ± 0.279	44.05 ± 0.23^S^	58.44 ± 0.98
7	35	7.212 ± 0.013^S^	8.311 ± 0.015	0.0661 ± 0.0037	0.0473 ± 0.0031	1.039 ± 0.006^S^	0.635 ± 0.019	78.88 ± 1.44^S^	95.49 ± 1.40
**Winery E**									
0	25	2.148 ± 0.003	2.318 ± 0.161	0.1603 ± 0.0021	0.1713 ± 0.0099	1.252 ± 0.0035	1.231 ± 0.0095	–	–
3.5	25	2.597 ± 0.016^S^	3.695 ± 0.109	0.1180 ± 0.0016	0.1343 ± 0.0053	1.186 ± 0.005	1.125 ± 0.035	41.47 ± 0.10^S^	57.86 ± 0.21
7	25	4.051 ± 0.003^S^	6.372 ± 0.063	0.06499 ± 0.0018	0.08217 ± 0.0302	1.043 ± 0.008	1.018 ± 0.142	75.15 ± 0.12^S^	87.19 ± 1.46
0	30	1.456 ± 0.156	1.958 ± 0.065	0.1924 ± 0.0055^S^	0.1584 ± 0.0042	1.254 ± 0.013	1.139 ± 0.001	–	–
3.5	30	2.008 ± 0.075^S^	3.069 ± 0.067	0.1161 ± 0.0003^S^	0.0998 ± 0.0030	1.109 ± 0.008^S^	0.947 ± 0.008	34.07 ± 0.58^S^	51.87 ± 0.49
7	30	4.715 ± 0.072	5.407 ± 0.586	0.0392 ± 0.0015	0.03448 ± 0.0005	2.233 ± 0.033^S^	1.295 ± 0.177	79.29 ± 0.43	87.89 ± 2.05
0	35	0.7815 ± 0.020^S^	1.686 ± 0.116	0.1171 ± 0.0016	0.1399 ± 0.0052	1.147 ± 0.014	1.107 ± 0.024	–	–
3.5	35	1.450 ± 0.061^S^	3.015 ± 0.097	0.07534 ± 0.0051	0.08448 ± 0.0018	0.932 ± 0.051	0.8141 ± 0.021	47.08 ± 0.43^S^	53.24 ± 0.19
7	35	2.177 ± 0.452	3.446 ± 0.814	0.0350 ± 0.0038^S^	0.007538 ± 0.010	0.4133 ± 0.009^S^	0.1737 ± 0.007	85.05 ± 1.35^S^	95.11 ± 1.28
**Winery F**									
0	25	1.516 ± 0.015	1.582 ± 0.096	0.1610 ± 0.0009	0.1613 ± 0.0017	1.229 ± 0.002	1.244 ± 0.008	–	–
3.5	25	2.547 ± 0.003^S^	3.450 ± 0.017	0.1250 ± 0.0015	0.1220 ± 0.0021	1.105 ± 0.007	1.020 ± 0.033	13.20 ± 1.08^S^	36.41 ± 1.26
7	25	4.057 ± 0.043^S^	5.597 ± 0.347	0.0925 ± 0.0029	0.1145 ± 0.0123	0.890 ± 0.003^S^	0.733 ± 0.004	41.28 ± 0.82^S^	85.72 ± 1.61
0	30	1.623 ± 0.012	1.712 ± 0.017	0.1880 ± 0.0022	0.1972 ± 0.0016	1.223 ± 0.005	1.175 ± 0.024	–	–
3.5	30	1.741 ± 0.132^S^	2.726 ± 0.031	0.1226 ± 0.0051	0.1225 ± 0.0010	1.012 ± 0.017^S^	0.8961 ± 0.007	11.48 ± 0.08^S^	48.16 ± 1.66
7	30	3.273 ± 0.043	4.957 ± 0.755	0.08269 ± 0.0222	0.0808 ± 0.0235	0.987 ± 0.058	0.948 ± 0.348	50.83 ± 0.19^S^	91.62 ± 1.24
0	35	0.939 ± 0.001	1.424 ± 0.042	0.1686 ± 0.0003	0.1717 ± 0.0010	1.187 ± 0.007^S^	0.9929 ± 0.002	–	–
3.5	35	1.905 ± 0.035^S^	2.570 ± 0.084	0.1206 ± 0.0070	0.1185 ± 0.0079	0.7066 ± 0.016^S^	0.5607 ± 0.027	13.21 ± 2.20^S^	59.33 ± 0.38
7	35	3.306 ± 0.466	4.879 ± 0.548	0.0430 ± 0.0061	0.0227 ± 0.0020	0.6424 ± 0.033^S^	0.3017 ± 0.035	47.02 ± 0.86^S^	98.09 ± 0.99

The growth parameters were calculated using Gompertz function (GraphPadPrism software), the inhibition percentages to ethanol at different temperatures were calculated using the following formula: = [1 – (Area under the growth curve in presence of ethanol at a 25, 30 or 35°C/Area under the curve without ethanol at a 25, 30, or 35°C)]^*^100. All the results are expressed as mean ± standard deviation; S, significant different (t-Test; p < 0.05); n.f., no significant fit.

**Figure 4:**
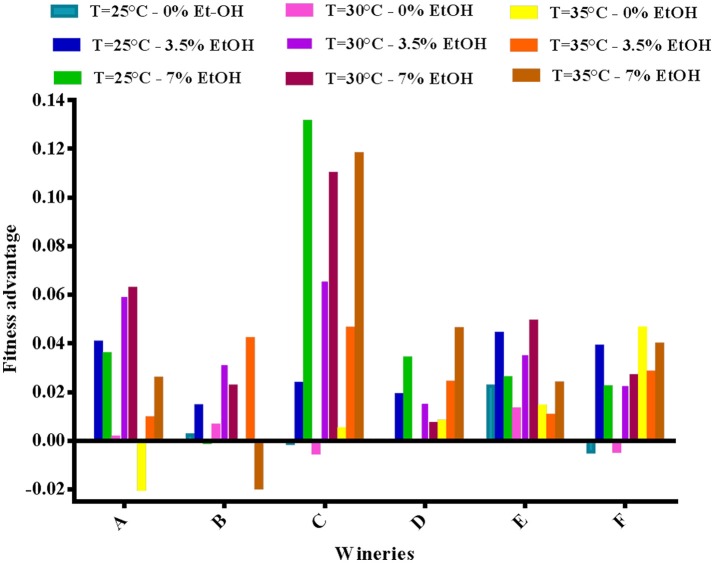
Fitness advantage at different concentrations of ethanol and temperatures calculated for each pair of HF/LF-*S. cerevisiae* strains from the six different wineries (A, B, C, D, E, and F), considering the average growth rate calculated between 0 and 8 h in synthetic medium.

Finally, the fitness advantage between the HF and LF strains was calculated for each winery taking into account the average growth rate between 0 and 8 h (v) using the mathematical formula reported above. As shown in Figure 4, the advantage of HF-*S. cerevisiae* strains was pointed out in most cases, with few exceptions (5 in total).

### Theoretical time required to achieve dominance of HF on LF-*S. cerevisiae* strains

**Figure 5:**
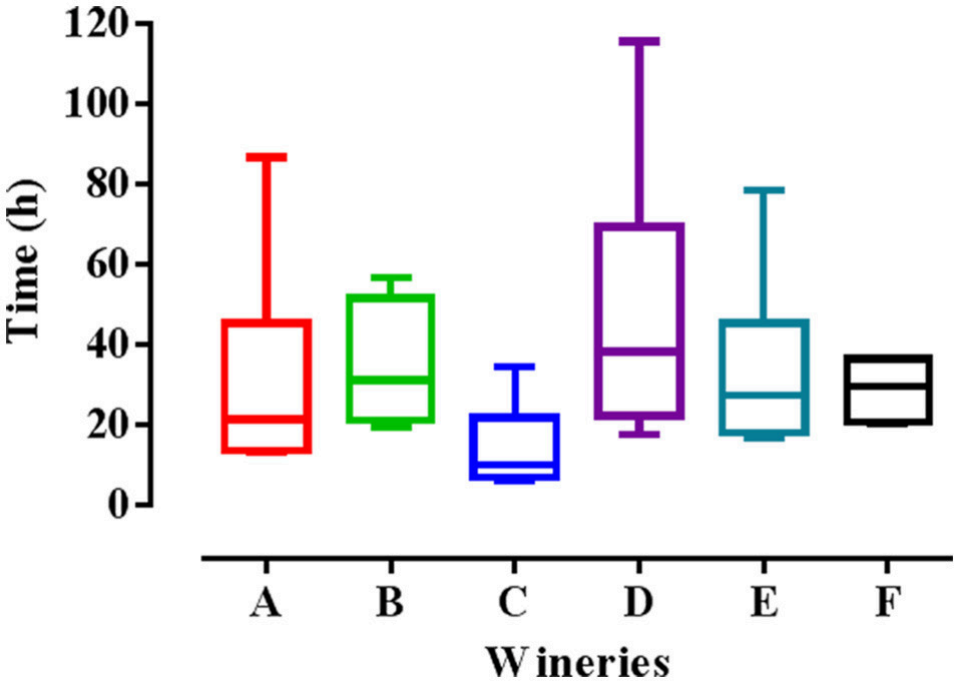
Theoretical time required by HF-*S. cerevisiae* strains to dominate on LF-*S. cerevisiae* strains in the six wineries studied (A, B, C, D, E, and F).

Fitness advantage in a specific competitive environment can explain why a given strain outcompetes another. Therefore, the values of fitness advantage reported in Figure 4 can be used to calculate the theoretical time (*t*) needed for HF-strains to dominate on LF-strains. The equation to calculate “*t*” was developed by Hartl and Clark (1997) and recently were used by Goddard (2008) and García-Ríos et al. (2014):

$$\begin{array}{l} t=\frac{1}{m}\ln\frac{ptq0}{qtp0} \end{array}$$

where “*m*” was the fitness advantage, “*p*” the frequency of HF-*S. cerevisiae* strains, “*q*” the frequency of LF-*S. cerevisiae* strains. In particular, *p*0 and *q*0 were the initial frequencies, while *pt* and *qt* were the final frequencies. The initial frequencies of both HF and LF-strains were imposed at 0.50, while the final frequencies were 0.90 and 0.10 for HF and LF-strains, respectively. In Figure 5 are reported the theoretical times required in each winery to achieve dominance of HF-*S. cerevisiae* on LF-strains. Theoretically, the assayed HF-*S. cerevisiae* strains takes an average of 14 to almost 50 h to dominate on LF-*S. cerevisiae* strains according to the winery considered. These theoretical values were in agreement with the experimental data obtained from laboratory-scale co-fermentations carried out by one HF and one LF strain that were inoculated in synthetic must at the same initial concentrations corresponding to frequencies at 0.50. Indeed, in each of six co-fermentations the HF-*S. cerevisiae* strain dominated on LF-strain within the first 24 h.

## Discussion

In spontaneous wine fermentation, different yeast species as well as various strains of the same species, usually coexist interacting with each other and the environmental conditions (Albergaria and Arneborg, 2016; Ciani et al., 2016; Morrison-Whittle and Goddard, 2018). Since during the alcoholic fermentation progress many changes occur in grape must becoming wine, the environmental conditions turn out to be more selective, and different yeast species and strains undergo sequential substitution in relation to their fitness for such harsh conditions (Bisson, 2012; Perrone et al., 2013; Williams et al., 2015; Ciani et al., 2016; Brice et al., 2018; Henriques et al., 2018). Different studies have raised evidence that the dominance of *S. cerevisiae* on non-*Saccharomyces* yeast species, that usually takes place in the first stages of spontaneous wine fermentation, is dependent on, not only higher tolerance to ethanol, but also on temperature (Goddard, 2008; Salvadó et al., 2011; Alonso del-Real et al., 2017), and other factors such as cell-to-cell contact mechanism (Nissen and Arnebor, 2003). On the other hand, few studies have investigated the dominance of *S. cerevisiae* strains during spontaneous or induced wine fermentation (Perrone et al., 2013; García-Ríos et al., 2014; Pérez-Torrado et al., 2017).

In this work, the influence of ethanol and temperature on the dominance of different *S. cerevisiae* strains, occurring in several spontaneous alcoholic fermentations carried out at industrial level in six wineries in Tuscany (Italy), was assayed by using the concept of fitness advantage (García-Ríos et al., 2014). The predominant *S. cerevisiae* strains were differentiated by RFLP-mtDNA method and according to their isolation frequency. The results obtained, by analyzing 637 isolates, confirmed the genetic polymorphism expected for *S. cerevisiae* population in spontaneous wine fermentations and the high variability between the isolation frequencies of different strains (Bisson, 2012; Schuller et al., 2012; Tofalo et al., 2013). In particular, independently of the grape variety, five out six wineries considered, showed only one predominant *S. cerevisiae* strain, with an isolation frequency ranging from 32 to 74%, while a variable number of strains (from 4 to 14) was characterized by an isolation frequency lower than 10%. These finding were consistent with those reported by other Authors (Versavaud et al., 1995; Gutiérrez et al., 1997; Egli et al., 1998; Sabate et al., 1998) although in some cases *S. cerevisiae* strains predominating the fermentative process, were not found (Vezinhet et al., 1992; Torija et al., 2001). In agreement with other studies (Versavaud et al., 1995; Barrajón et al., 2010), the indigenous *S. cerevisiae* strains were differentiated in two groups: strains at high frequency (HF) or “predominant” and strains at low frequency (LF) or “secondary” strains. Moreover, our results demonstrated that the *S. cerevisiae* strains dominating spontaneous wine fermentations were not related to the grape variety used to perform alcoholic fermentations; instead, they were representative of different wineries, strengthening the idea of the occurrence of yeast strains possessing better fitness to the specific winemaking conditions used in each winery (Cocolin et al., 2004). Probably, during the usual cellar operations, yeast strains spread throughout the environment and those that were better adapted to certain conditions occurred at higher frequencies, becoming the dominant yeast strains in the winery. In the literature, in some cases *S*. c*erevisiae* strains were found to be capable of dominating the alcoholic fermentation in all vats of the same winery, independently of the grapevine cultivar (Frezier and Dubourdieu, 1992; Guillamón et al., 1996), whereas other times the yeast strains were found to be specific for each grape variety (Blanco et al., 2006; Schuller et al., 2012).

In any case, cluster analysis with the Dice coefficient and the UPGMA method grouped the profiles of predominant (HF) and secondary (LF) *S. cerevisiae* strains in clusters according to the winery where they came from. Furthermore, the yeast commercial strains assayed in this study, chosen because they are the most frequently used in Tuscany as starter cultures, grouped into a distinct cluster indicating that they were significantly different from the indigenous strains, probably because of they were isolated from French oenological areas. Mercado et al. (2011), by using two molecular methods (RFL-mtDNA and *interdelta* PCR) observed a clear separation between *S. cerevisiae* strains isolated from vineyard and commercial strains, while in other study on cellar-associated *S. cerevisiae* population structure “only 7% of cellar strains were found to be related to commercial strains usually used” as starter cultures (Börlin et al., 2016). On the contrary, Martiniuk et al. (2016) found that commercial and commercial-related yeasts occurred in spontaneous fermentations of a Canadian winery, although they did not dominate the *S. cerevisiae* populations that were unrelated to commercial strains present in the same fermentations. Concerning this work, it should be emphasized that five out of six wineries here taken into account never used commercial yeast strains and only the winery E used the S1 strain as starter some years before the survey.

The occurrence of specific *S. cerevisiae* strains in each winery supports the potential role of these microorganisms in determining distinctive wine characteristics and their selection could represent a resource to contribute in preserving the typicality of wines (Vezinhet et al., 1992; Augruso et al., 2008; Aponte and Blaiotta, 2016; Bokulich et al., 2016). Recent studies suggested the concept of “the so-called microbial *terroir*” demonstrating that indigenous yeast strains can be associated to a given viticultural region (Bokulich et al., 2016; Morrison-Whittle and Goddard, 2018). However, according to our results, specific *S. cerevisiae* strains seem to be representative of single winery rather than of an oenological area: three out six wineries (A, B, and C) were situated within 10 km radius, and showed *S. cerevisiae* grouped in three different *clusters*. Therefore, data suggested the idea of the “winery effect” or a microbial *terroir* at a smaller scale. Nevertheless, in order to assess the existence of certain relationship between indigenous *S. cerevisiae* strains and single winery, further surveys in consecutive years in the same wineries located in different oenological areas should be carried out.

The further step was addressed to confirm, in laboratory-scale fermentations, the dominant behavior, exhibited by *S. cerevisiae* strains at high frequency (HF) in the spontaneous alcoholic fermentations in each winery. Co-fermentations were carried out by inoculating at the same cell densities (10^4^ cell/mL) one HF-strain and one LF-strain coming from the six wineries, and the ability of one strain to dominate over another was assayed by using the RFLP-mtDNA method. The data obtained raised evidence that after 24 h in co-fermentations total yeast population reached values of 10^7^ CFU/mL and that in all our trials the “HF” *S. cerevisiae* strain occurred at frequency ranging from 70 to 87%, confirming the dominance behavior observed in industrial spontaneous fermentations in the six wineries. Other Authors (Barrajón et al., 2010; Perrone et al., 2013; Pérez-Torrado et al., 2017) that assayed the competition between indigenous “dominant” *S. cerevisiae* strains and commercial yeasts or between one “dominant” and one “non-dominant” strain by using co-fermentations, reported similar results. This dominance phenomenon has been mainly attributed to a cell-to-cell contact mechanism or microenvironment contact, conditions in which cells compete for space when are in high densities and in cell-to-cell contact, so that the non-dominant yeast strain arrests its growth (Ciani et al., 2016). Moreover, a differential sulphite production and resistance and the killer activity seemed to be involved in dominant behavior of the yeast strains (Perrone et al., 2013; Pérez-Torrado et al., 2017). It is noteworthy that no killer activity was detected in HF-strains assayed in this study and no significant differences in sulphite production were found (data not shown).

Nevertheless, the competition degree of each strain, which determine the capacity of one strain to out-compete another, is influenced by other factors including pH, temperature, ethanol, osmotic pressure, nitrogen available (Ciani et al., 2016). Indeed, our findings concerning the influence of ethanol and temperature on the growth performance and the fitness advantage of High frequency (HF) *S. cerevisiae* strains, support the important role that these two factors may play in determining the dominance of one strain over another in wine fermentations. By considering the single effect of ethanol on growth performance, “HF” strains showed significant lower inhibition percentages than “LF” strains although in the presence of different ethanol concentrations (from 3 to 7%). The inhibition percentages, calculated as reported by Arroyo-López et al. (2009, 2010), was an appropriate indicator of the overall yeast growth as this parameter was inversely related to the lag phase, but linearly related to both the maximum specific growth rate (μ_max_) and the maximum cell densities at the end of growth. Consequently, the fitness advantage, which according to Salvadó et al. (2011) represents the difference in μ_max_ between competitors for a specific environmental condition, resulted higher for “HF” strains, suggesting their better adaptability to increasing ethanol concentrations in comparison with “LF” strains. However, this capability resulted to be a strain-dependent characteristic as the fitness advantage showed values ranging from 1 to 6% per hour and from 1 to 10% per hour in the presence of 3 and 5% ethanol, respectively. Indeed, each *S. cerevisiae* strains may display different stress responses to ethanol as the effects of increasing ethanol concentrations on the yeast cell include different changes such as in membrane composition and in gene expression, synthesis of heat shock proteins, increases in chaperons proteins etc. (Ding et al., 2009). Recently, a study aimed to assess fitness advantages of four commercial wine yeast strains has stressed that fermentation temperature might be an important factor in determining the dynamics of the *S. cerevisiae* strain population (García-Ríos et al., 2014). In fact, ethanol and high temperature affect synergistically the membrane integrity and permeability causing a decrease in the growth of yeast populations (Alexandre et al., 1994; Albergaria and Arneborg, 2016). The data related to the combined effect of increasing ethanol concentrations and different temperatures on the growth performance and the fitness advantages of six couple of HF and LF-*S. cerevisiae* here considered, confirmed that these two factors could play an important role in niche construction favoring some strains of *S. cerevisiae* compared to others in wine fermentation. According to some studies, the competitive advantage of *S. cerevisiae* on non-*Saccharomyces* yeasts in spontaneous alcoholic fermentations seems to be related to both ethanol and temperature adaptation (Goddard, 2008; Salvadó et al., 2011; Ciani et al., 2016; Alonso del-Real et al., 2017). Therefore, similar competition mechanisms might be responsible for interaction among indigenous *S. cerevisiae* strains. Our results proved that the six “HF” strains had always fitness advantage in comparison with relative LF strains when temperature was 25 or 30°C in the presence of ethanol concentrations of 3.5 and 7% v/v. These conditions typically occur in the early stages of alcoholic fermentations and suggest that they can affect the competition among different *S. cerevisiae* strains during the first 2 days of the fermentative process.

Taking into account values of fitness advantage obtained at different temperature and ethanol concentrations was calculated the hypothetical time needed for each “HF”-*S. cerevisiae* to achieve dominance on the relative “LF”-*S. cerevisiae* strain in a theoretical mixed population in which each strain was equally represented (50%) (García-Ríos et al., 2014). Results showed that assayed “HF”-*S. cerevisiae* strains took an average of 14 to almost 50 h to dominate on “LF”-*S. cerevisiae* strains based in relation to the winery where they originated.

In conclusions, these findings support the key role of ethanol and temperature in determining fitness advantage of some *S. cerevisiae* strains and contribute to the understanding of predominance of *S. cerevisiae* strains in spontaneous wine fermentations, even though other factor and or mechanisms can be involved. Moreover, these yeast strains could be exploited to develop new wine *starters* able to guarantee a high fermentative performance in grape musts even under stressful conditions and a positive metabolites production in the final wine (Bonciani et al., 2016). Recently, in order to achieve this goal, the construction of hybrid *S. cerevisiae* strains has been performed through selection programs based on the adaptive evolution strategy or a multi-phase approach (Bonciani et al., 2018), valuable tools to obtain improved and suitable yeast strains in the modern oenology.

## Author contributions

SG, DG, SM, and LG conceived and designed the experiments. SM contributed to perform chemical analysis. DG contributed to perform microbiological analysis and genotyping characterization of *S. cerevisiae* isolates. SG and LG contributed to statistical analysis and interpretation of data for the work, to draft the work and revising it. MV contributed to the revision of the work.

### Conflict of interest statement

The authors declare that the research was conducted in the absence of any commercial or financial relationships that could be construed as a potential conflict of interest.
